# Supplementary material for: Linking Groundwater to Surface Discharge Ecosystems: Archaeal, Bacterial, and Eukaryotic Community Diversity and Structure in Quebec (Canada)
Source: Microorganisms. 2023 Jun 27;11(7):1674. doi: 10.3390/microorganisms11071674 (PMC10384904; doi:10.3390/microorganisms11071674)
Supplement: Supplementary file 1 [file microorganisms-11-01674-s001.zip › microorganisms-2432147-supplementary/Table S1.pdf]

Table S1. List of groundwater site and surface water site names used throughout the study.

**Groundwater well**	**Surface water**
P_10, P_14, P_15, P_16	S_15 (lake)
P_20, P_27, P_36, P_41, P_75	S_20 (lake)
P_85, P_88, P_89	S_85 (lake)
P_81, P_82, P_83, P_84	S_82 (lake)
P_45, P_107, P_123	S_107 (lake)
P_178, P_192, P_193, P_194; P_200, P_203	S_203 (lake)
P_178, P_192, P_193, P_194; P_200, P_203	S_204 (lake)
P_157, P_158	S_156 (lake)
P_151, P_309	S_271 (stream)
P_134, P_152, P_153, P_154	S_154 (stream)
P_256, P_262, P_265, P_270, P_331, P_332, P_335	S_332 (river)
	S_57 (lake)
	S_73 (lake)
	S_142 (lake)
	S_171 (lake)
	S_348 (lake)
	S_310 (lake)
