# Supplementary material for: Linking Groundwater to Surface Discharge Ecosystems: Archaeal, Bacterial, and Eukaryotic Community Diversity and Structure in Quebec (Canada)
Source: Microorganisms. 2023 Jun 27;11(7):1674. doi: 10.3390/microorganisms11071674 (PMC10384904; doi:10.3390/microorganisms11071674)
Supplement: Supplementary file 1 [file microorganisms-11-01674-s001.zip › microorganisms-2432147-supplementary/Table S3.pdf]

Table S3. Homova analysis for the surface (S) and subsurface (SS) groups.

ARCHAEA

	BValue	P-value	SSwithin/(Ni-1)_values	
**SS-S**	0.0077347	<0.001*	0.484059	0.464893

BACTERIA

	BValue	P-value	SSwithin/(Ni-1)_values	
**SS-S**	0.272336	<0.001*	0.48703	0.389358

EUKARYOTE

	BValue	P-value	SSwithin/(Ni-1)_values	
**SS-S**	0.0846235	<0.001*	0.473992	0.417609

SS=subsurface
S=surface
