# Supplementary figures and images for: Linking Groundwater to Surface Discharge Ecosystems: Archaeal, Bacterial, and Eukaryotic Community Diversity and Structure in Quebec (Canada)
Source: Microorganisms. 2023 Jun 27;11(7):1674. doi: 10.3390/microorganisms11071674 (PMC10384904; doi:10.3390/microorganisms11071674)

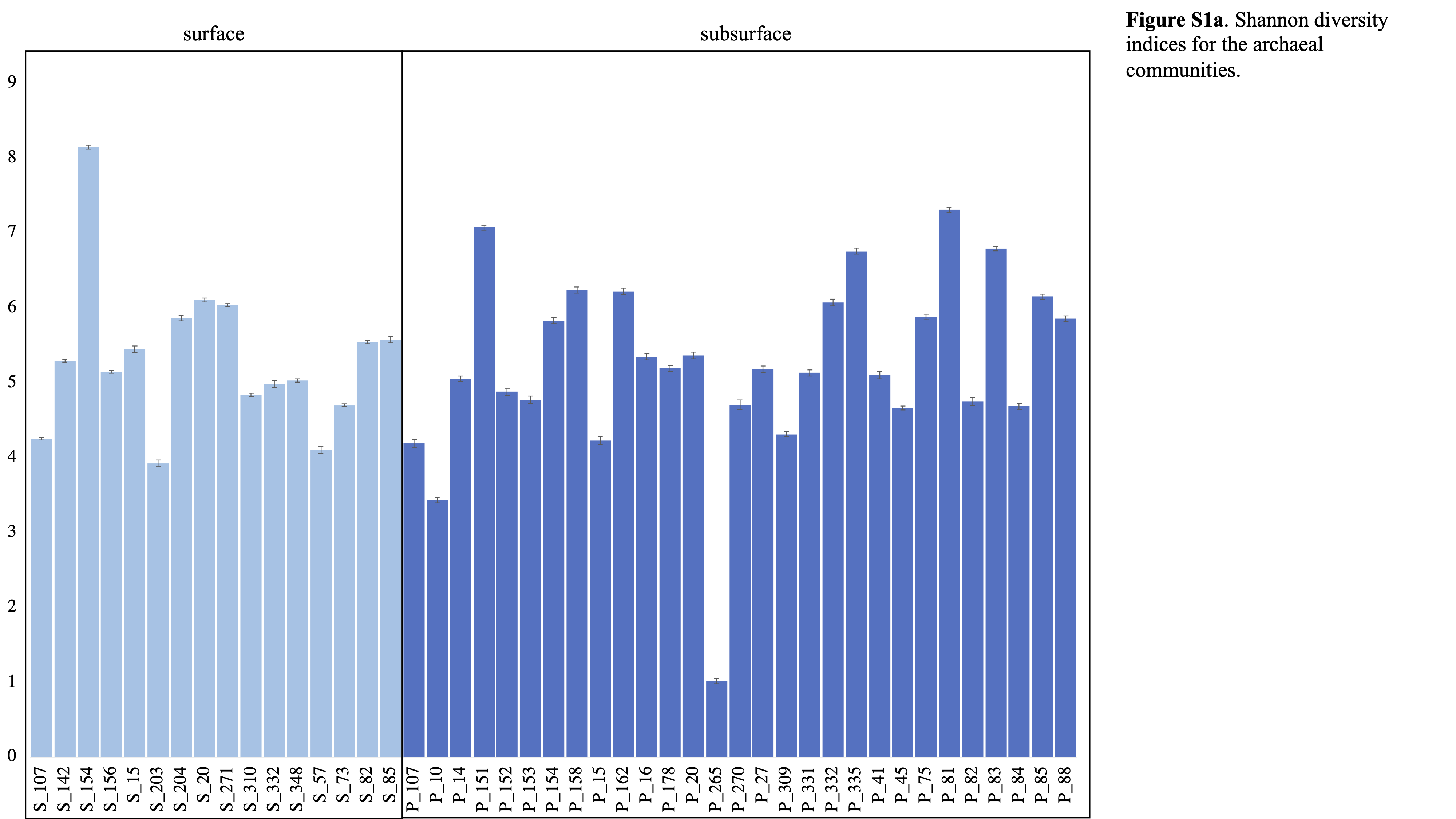

Supplement: Supplementary file 1 [file microorganisms-11-01674-s001.zip › microorganisms-2432147-supplementary/Figure S1a.tiff]

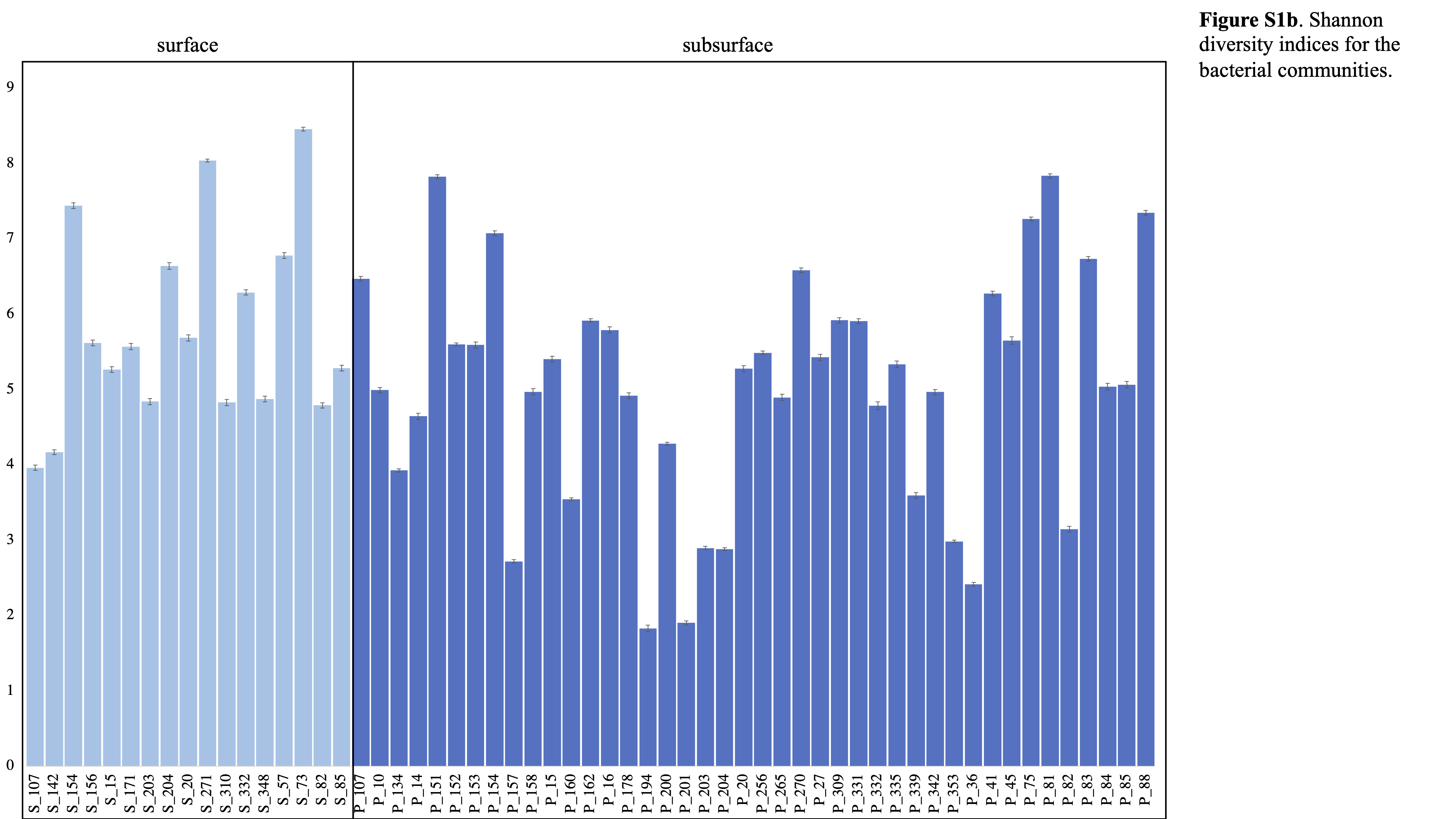

Supplement: Supplementary file 1 [file microorganisms-11-01674-s001.zip › microorganisms-2432147-supplementary/Figure S1b.tiff]

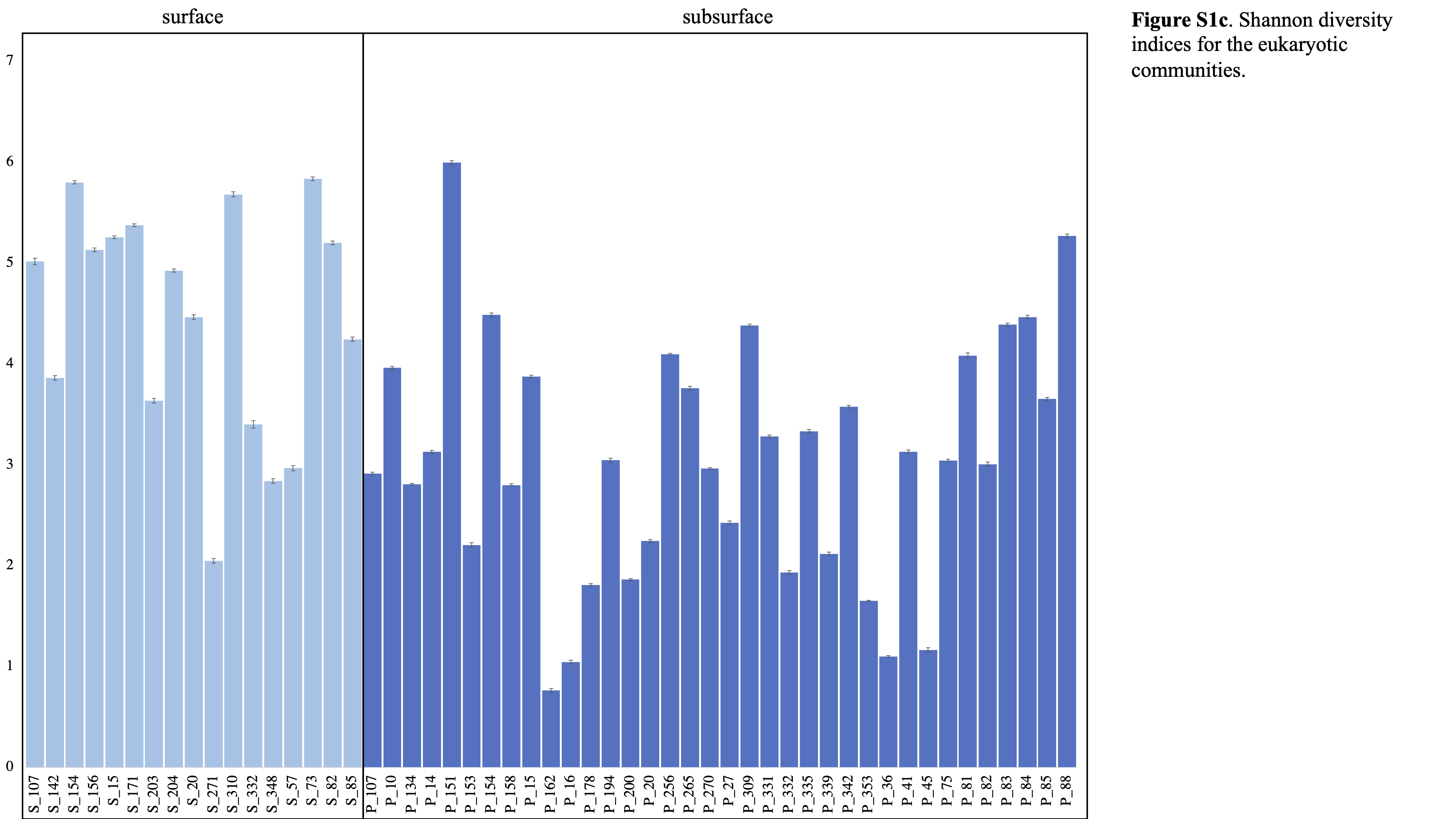

Supplement: Supplementary file 1 [file microorganisms-11-01674-s001.zip › microorganisms-2432147-supplementary/Figure S1c.tiff]

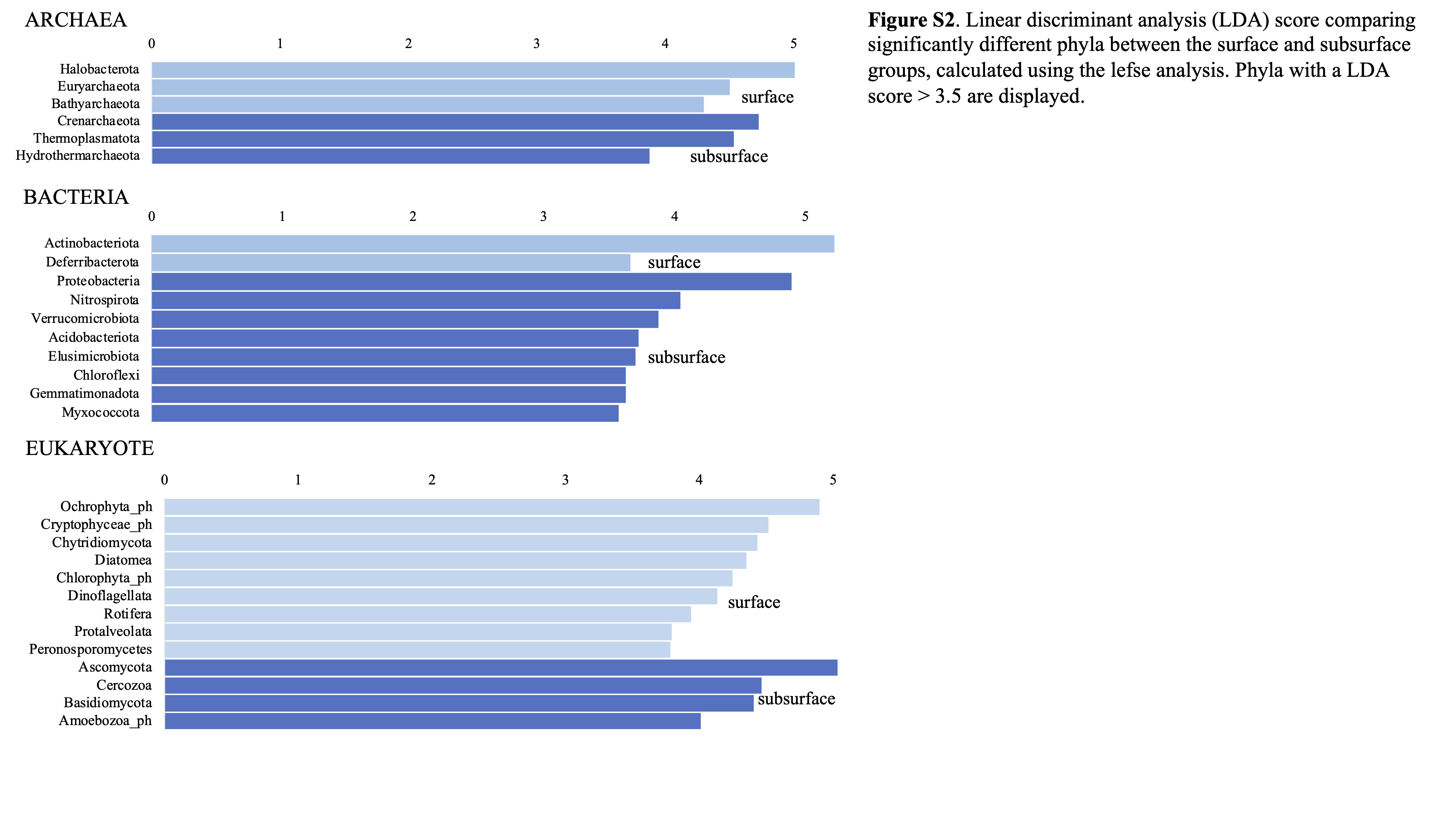

Supplement: Supplementary file 1 [file microorganisms-11-01674-s001.zip › microorganisms-2432147-supplementary/Figure S2.tiff]

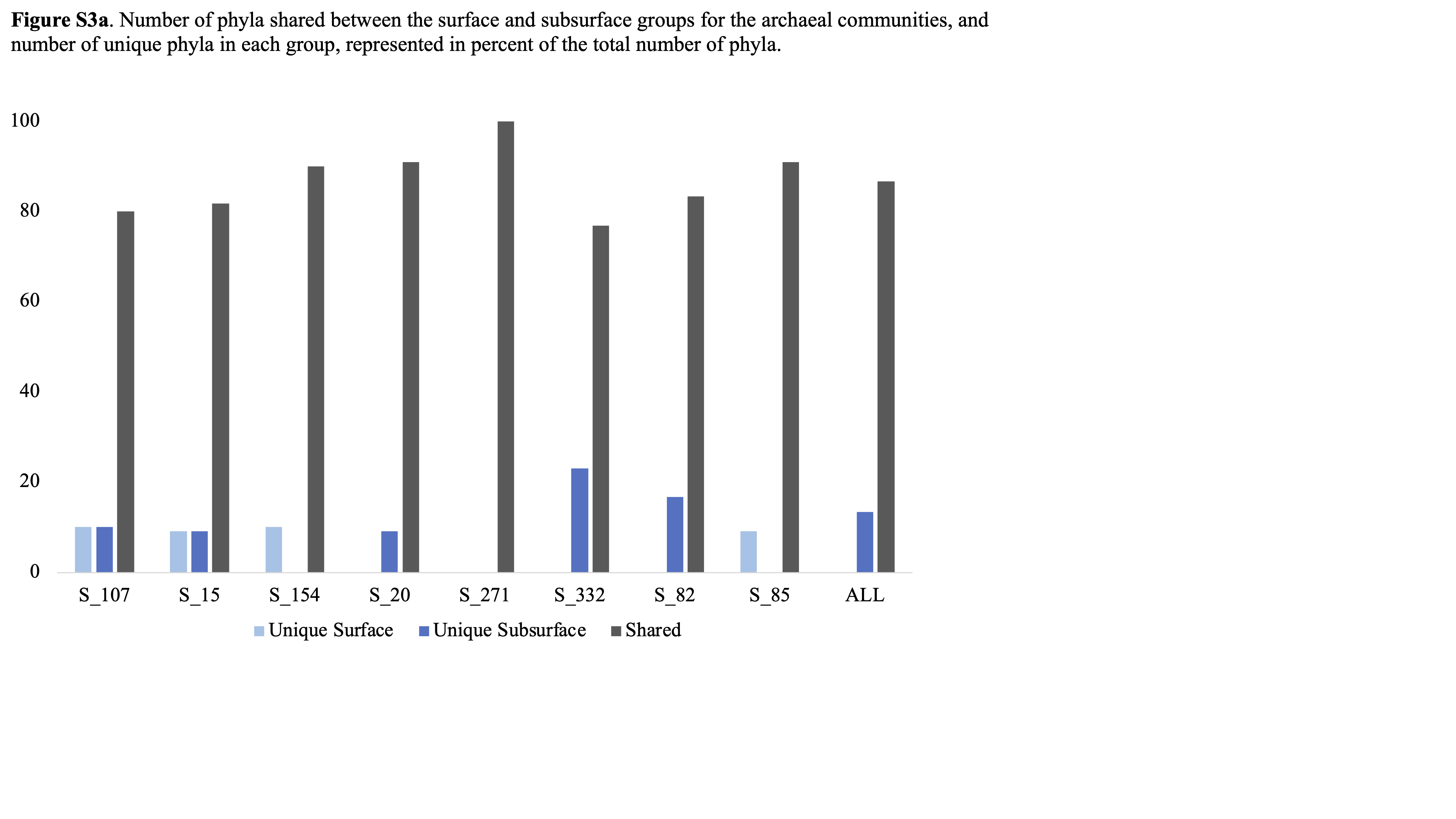

Supplement: Supplementary file 1 [file microorganisms-11-01674-s001.zip › microorganisms-2432147-supplementary/Figure S3a.tiff]

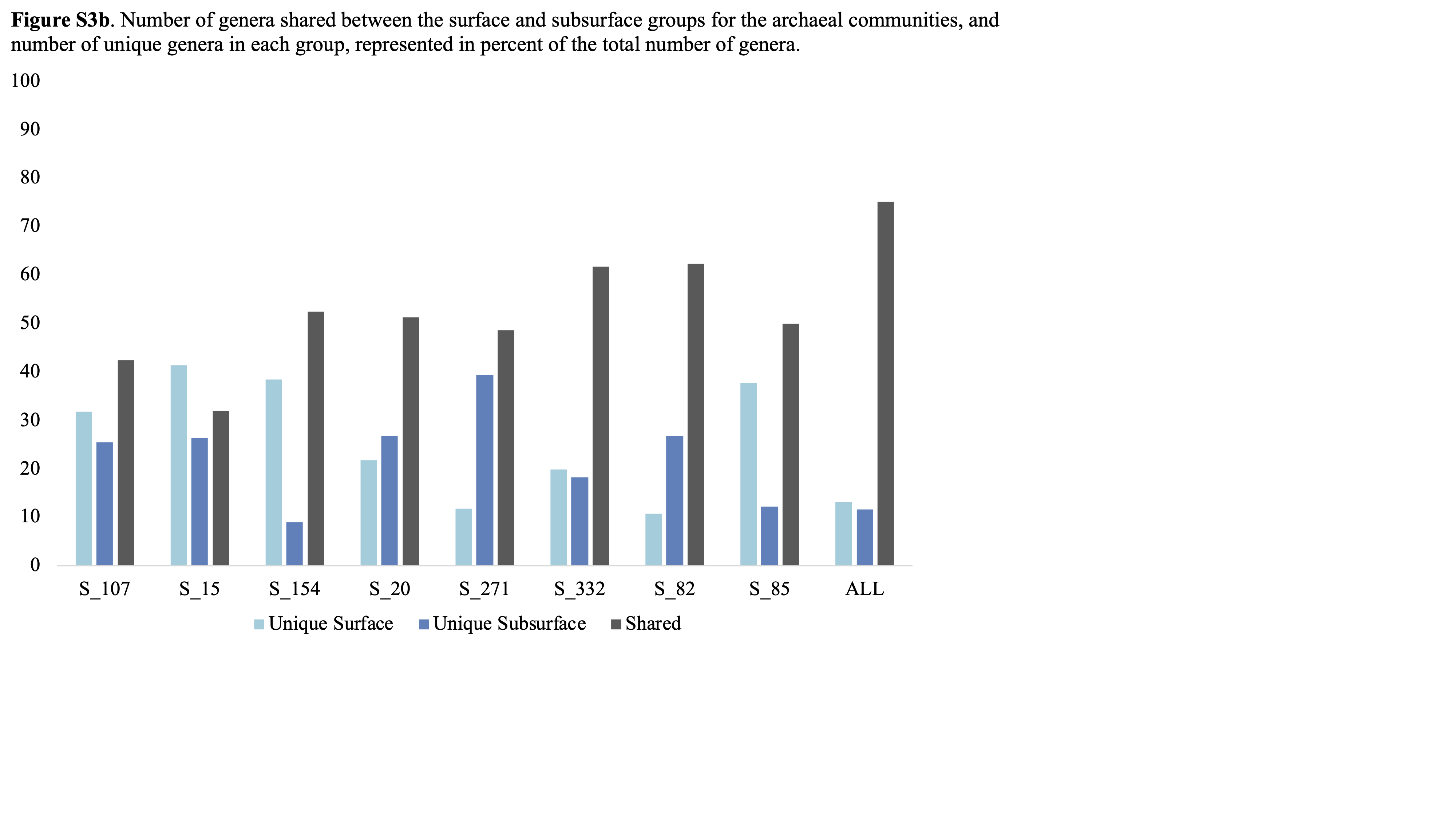

Supplement: Supplementary file 1 [file microorganisms-11-01674-s001.zip › microorganisms-2432147-supplementary/Figure S3b.tiff]

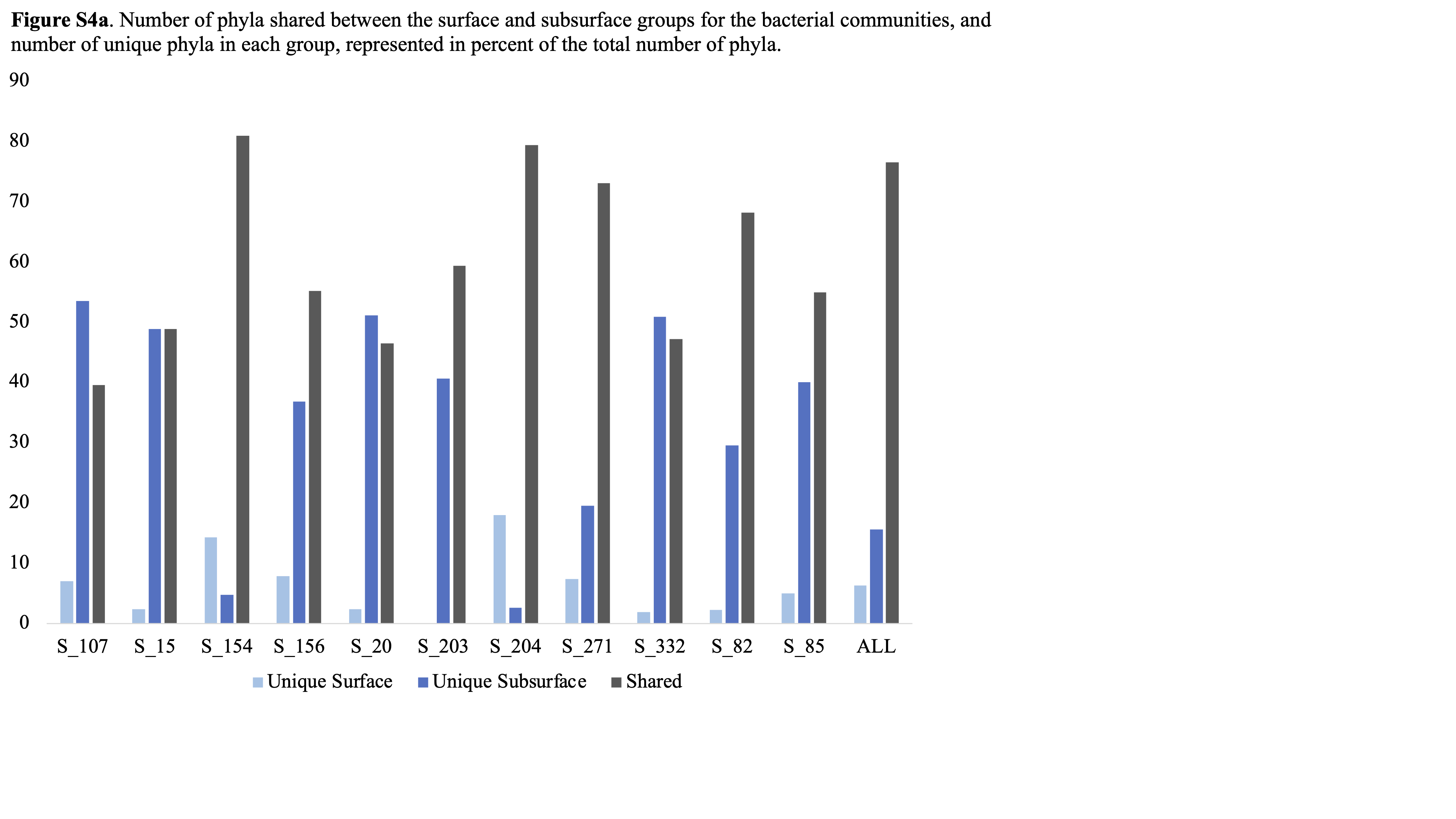

Supplement: Supplementary file 1 [file microorganisms-11-01674-s001.zip › microorganisms-2432147-supplementary/Figure S4a.tiff]

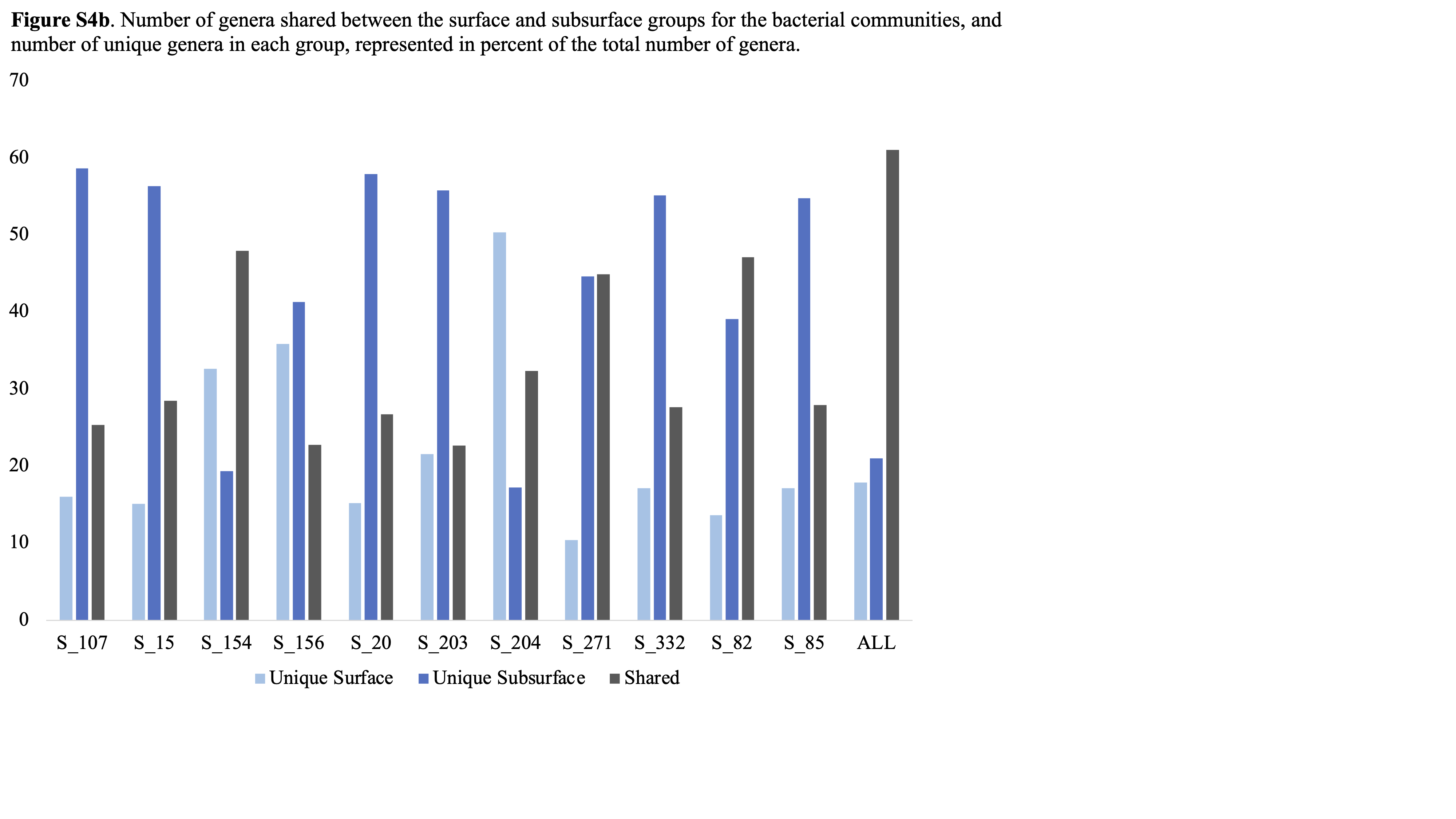

Supplement: Supplementary file 1 [file microorganisms-11-01674-s001.zip › microorganisms-2432147-supplementary/Figure S4b.tiff]

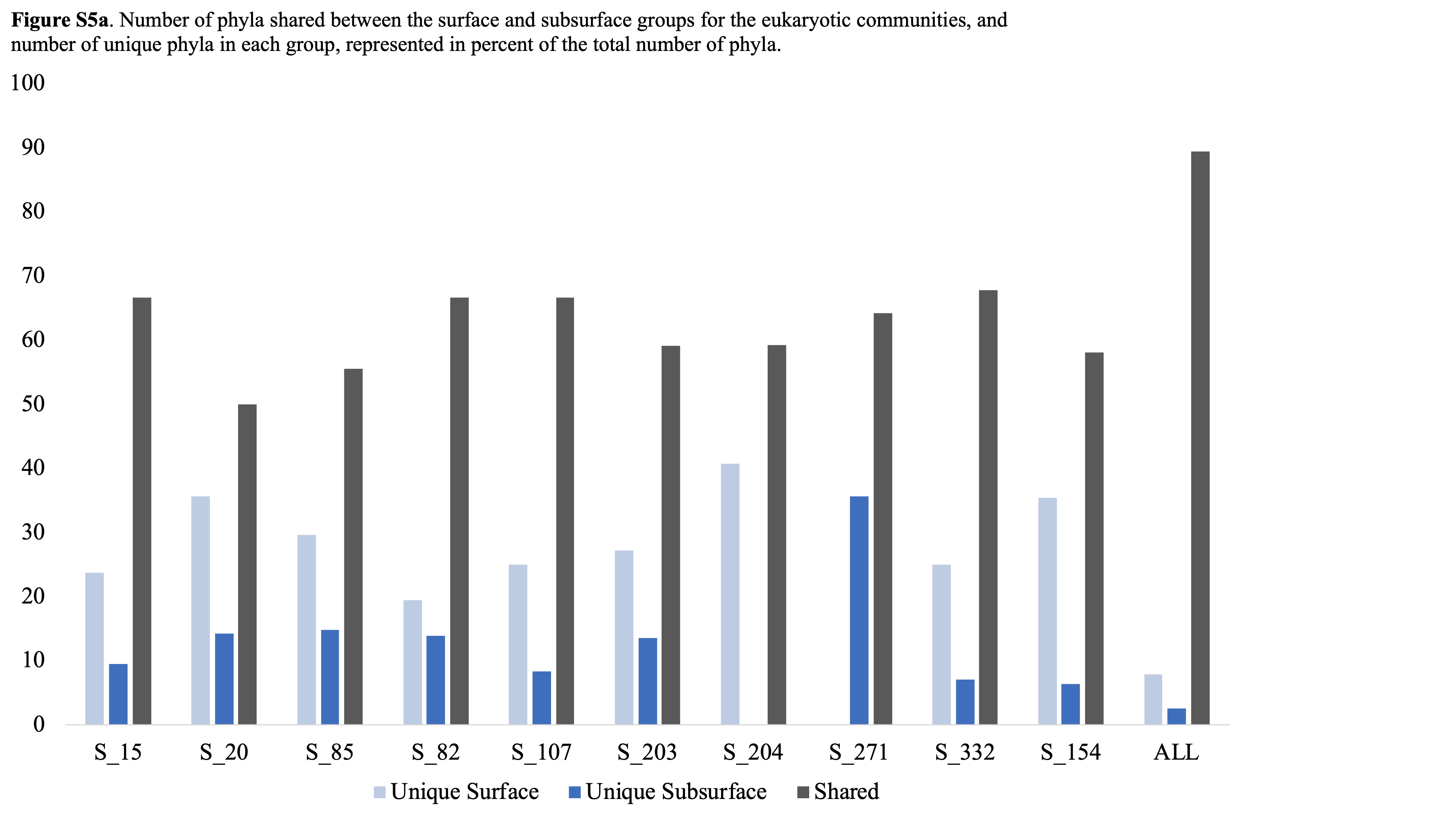

Supplement: Supplementary file 1 [file microorganisms-11-01674-s001.zip › microorganisms-2432147-supplementary/Figure S5a.tiff]

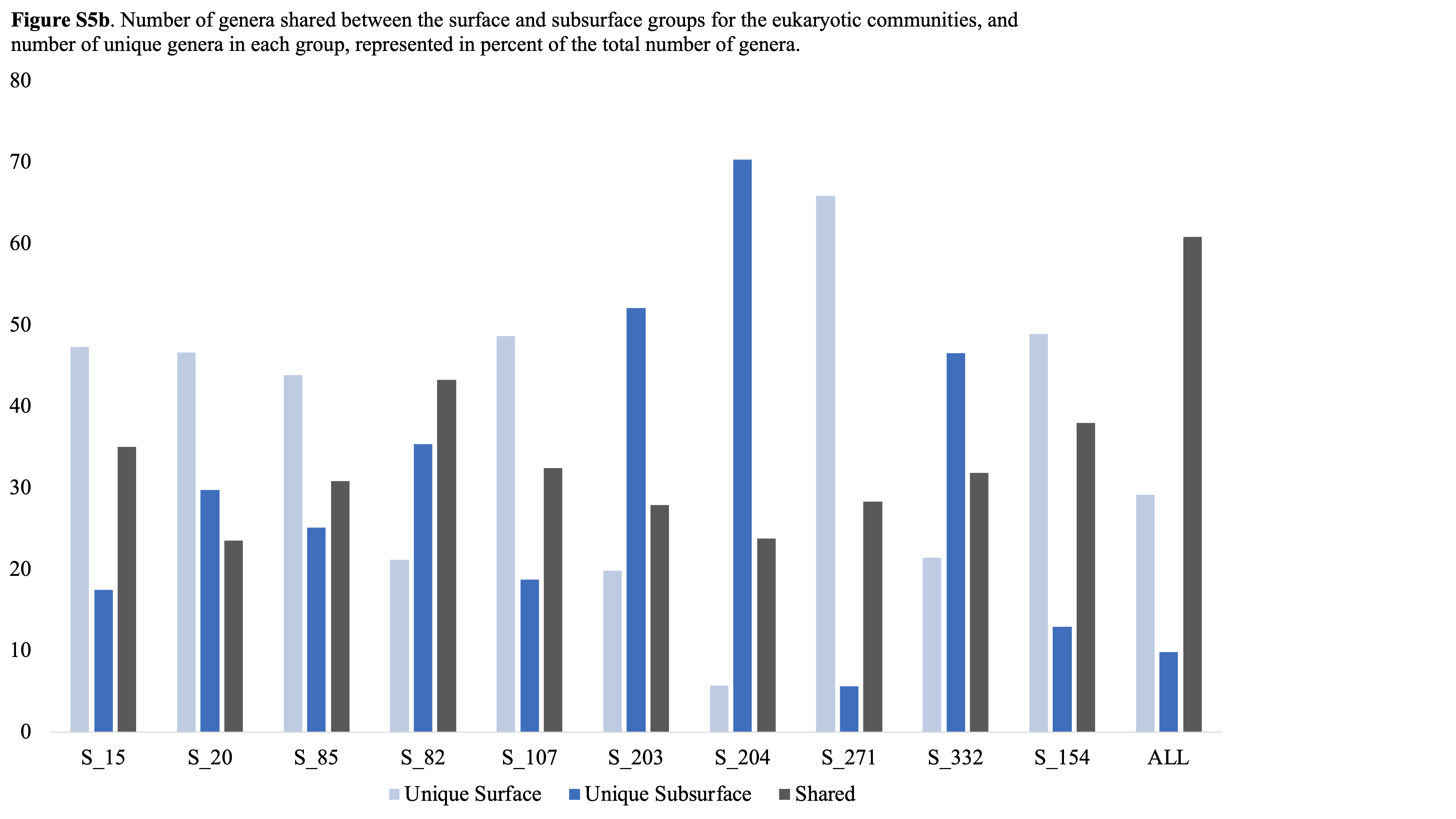

Supplement: Supplementary file 1 [file microorganisms-11-01674-s001.zip › microorganisms-2432147-supplementary/Figure S5b.tiff]

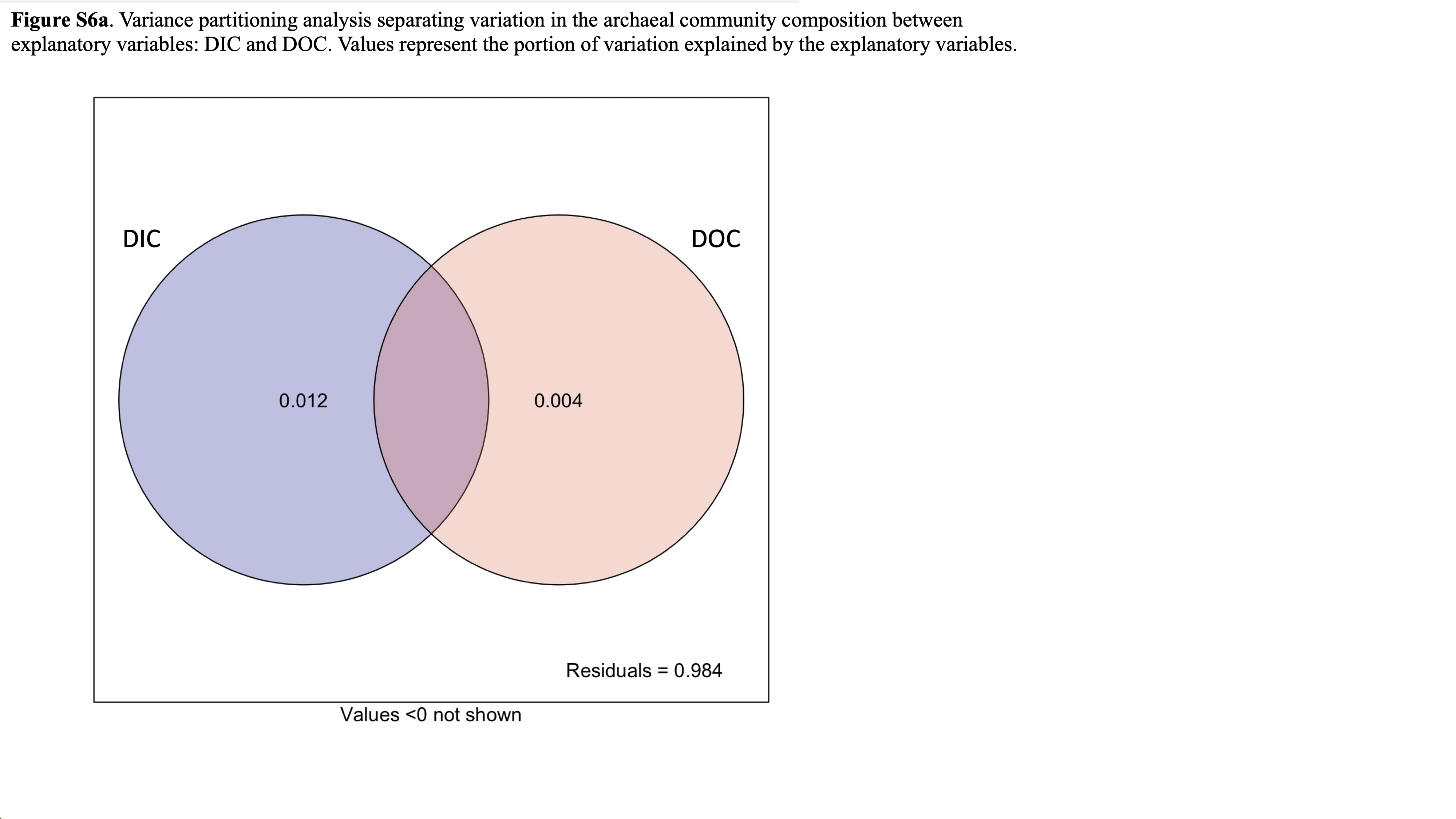

Supplement: Supplementary file 1 [file microorganisms-11-01674-s001.zip › microorganisms-2432147-supplementary/Figure S6a.tiff]

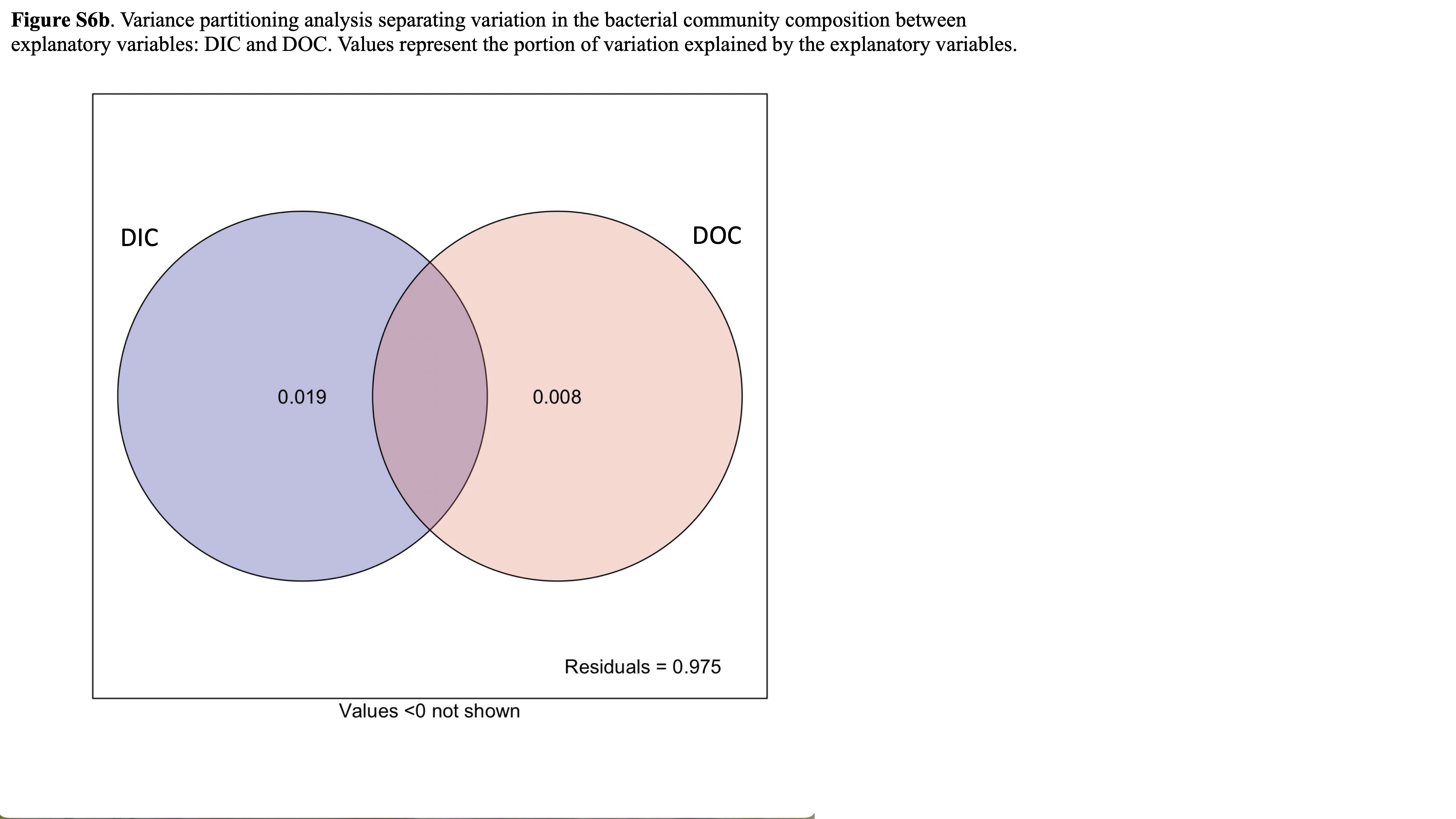

Supplement: Supplementary file 1 [file microorganisms-11-01674-s001.zip › microorganisms-2432147-supplementary/Figure S6b.tiff]

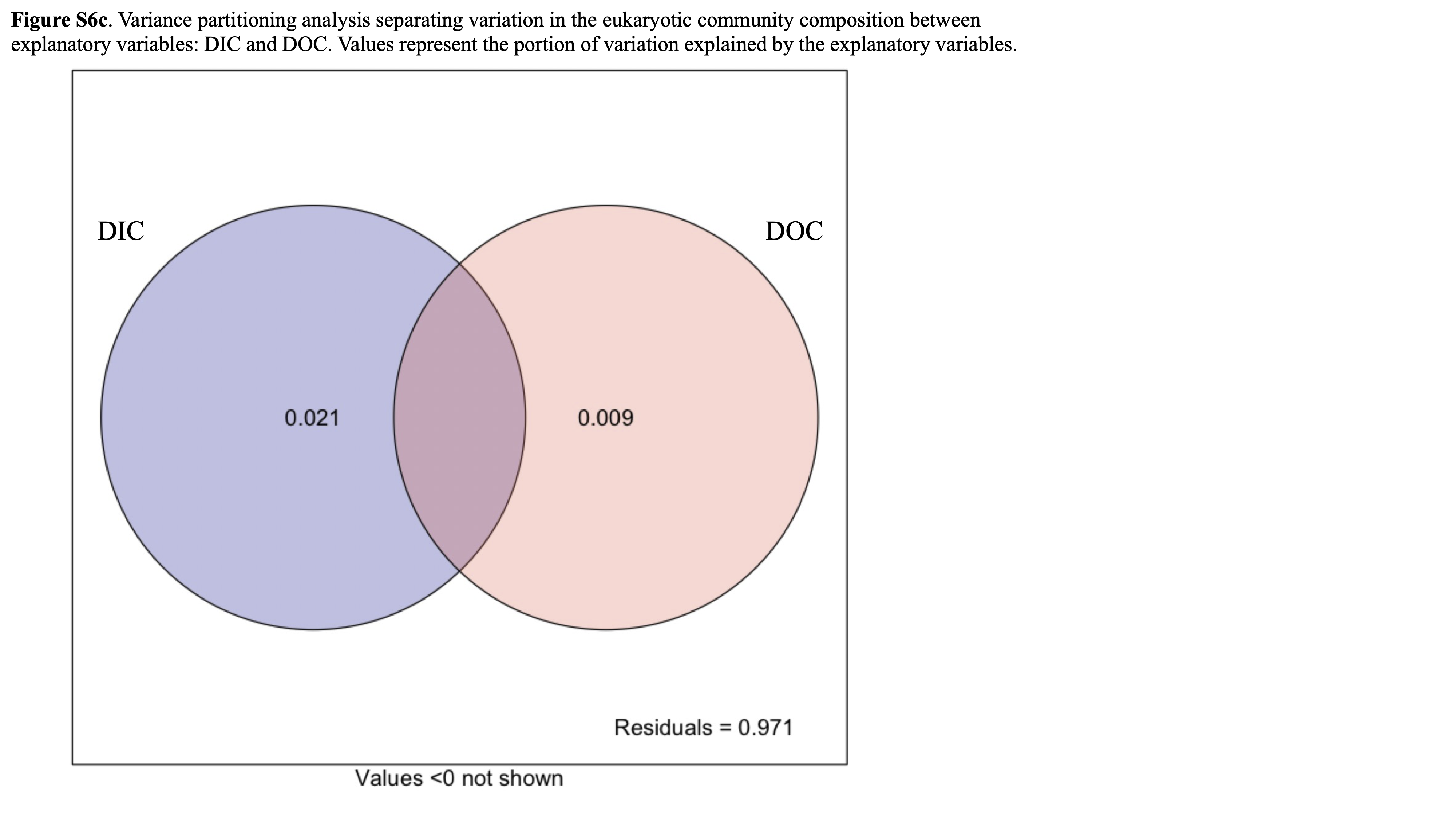

Supplement: Supplementary file 1 [file microorganisms-11-01674-s001.zip › microorganisms-2432147-supplementary/Figure S6c.tiff]
